# Imaging and pathological comparison of inflammatory pseudotumor-like follicular dendritic cell sarcoma of the spleen: A case report and literature review

**DOI:** 10.3389/fsurg.2022.973106

**Published:** 2022-09-05

**Authors:** Fuxing Chen, Junqiang Li, Pingkun Xie

**Affiliations:** ^1^ Department of Radiology, Beilun District People’s Hospital, Ningbo, China; ^2^ Department of Pathology, Beilun District People’s Hospital, Ningbo, China

**Keywords:** spleen tumor, follicular dendritic cell sarcoma, tomography, x-ray computed, magnetic resonance imaging

## Abstract

**Background:**

Inflammatory pseudotumor-like follicular dendritic cell sarcoma (IPT-like FDCS) is a rare subtype of follicular dendritic cell sarcoma (FDCS) that is mainly located in the liver and spleen (1). Splenic IPT-like FDCS is a rare low-grade malignancy with non-specific clinical manifestations and laboratory findings. Herein, we reported the pathological and imaging features of a case with splenic IPT-like FDCS.

**Case presentation:**

A 57-year-old woman was found to have a mass in the spleen during a physical examination and was hospitalized for further treatment. Her laboratory results were within the normal range. Unenhanced and contrast-enhanced computed tomography scans of the whole abdomen showed a round mass in the spleen, with a diameter of about 5 cm. After further examination with enhanced MRI, a provisional diagnosis of splenic hemangioma or splenic hamartoma was made. The patient underwent splenectomy, and the pathological diagnosis was splenic IPT-like FDCS. No tumor recurrence or metastasis was found during the 1-year follow-up after the operation.

**Conclusions:**

Herein, we reported a case of splenic IPT-like FDCS. Although the clinical examination and laboratory examination lack specificity, the imaging of this case showed that the lesion was a solid mass with progressive enhancement, and the central scar showed the characteristics of delayed enhancement, which facilitated the diagnosis.

## Background

Inflammatory pseudotumor (IPT)-like follicular dendritic cell sarcoma (FDCS) is a rare low-grade malignancy that occurs most often in the liver or spleen (1). The pathogenesis and causes remain unclear, but EBV infection is considered one of the most important etiologies of this tumor (2), as almost all cases are EBER positive. Despite its slow growth and low malignancy, the recurrence rate is approximately 10% (3). Due to histomorphological similarity, this tumor is often confused with inflammatory myofibroblastic tumors, such as benign reactive IPT and inflammatory myofibroblastic tumors (4–6). Due to the lack of imaging and clinical characteristics, preoperative diagnosis is difficult and mainly relies on the pathological diagnosis. Herein, we described the imaging characteristics and comparative pathological analysis of a case of splenic IPT-like FDCS.

## Case description

**Figure 1:**
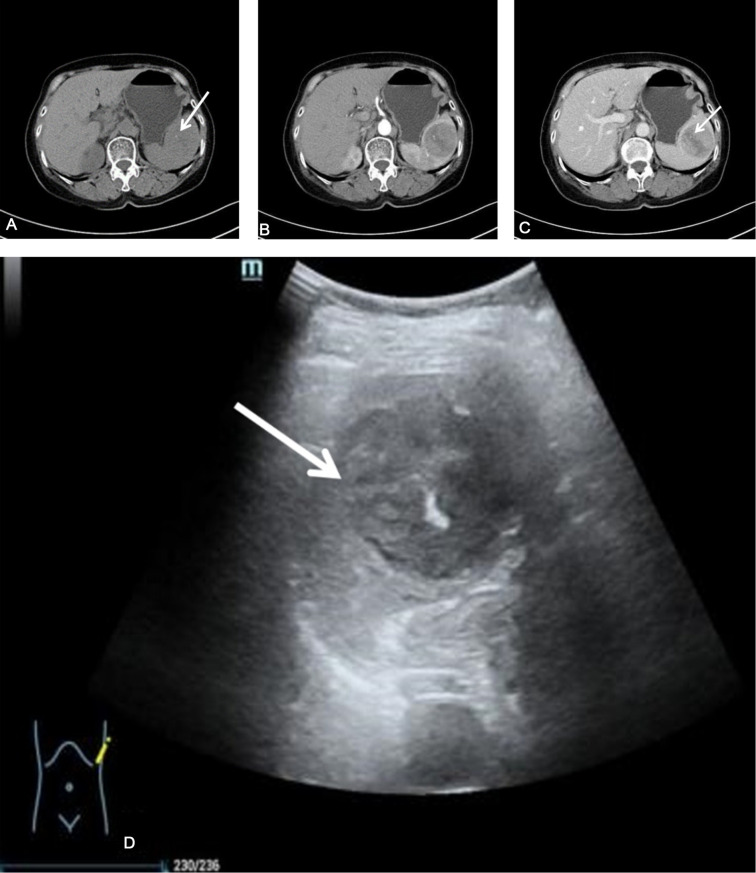
The patient's splenic CT scan. (**A**) Plain CT scan showed that the spleen was a round mass shadow in the parenchyma, with punctate calcifications (white arrows). The normal parenchyma was poorly demarcated. (**B,C**) Contrast-enhanced CT showed that the tumor enhancement was weaker than that of the normal spleen, with clear borders, and a strip-like cleft-like non-enhanced area (white arrow). (**D**) B-ultrasound showed a heterogeneous low echo in the spleen, with a spot-like strong echo (white arrow). CT, Computed Tomography.

The patient was a 57-year-old female who underwent physical examination seven days ago at the Department of Radiology, Beilun People's Hospital, Ningbo, Zhejiang Province. The ultrasonographic findings were as follows: Inhomogeneous hypoechoic mass with calcification in the spleen, hemangioma requiring excision, and further examination was recommended (Figure 1D). The patient was hospitalized for further treatment. The patient was healthy in the past, and the spleen was not palpable under the ribs on physical examination. The patient's biochemical indexes and liver and kidney function electrolyte indexes were within the normal range. Routine blood, urine, and fecal tests were also within normal ranges. Blood tumor markers CEA, CA-199, CA-125, and AFP were all normal, and coagulation function, electrocardiogram and chest x-ray were normal.

**Figure 2:**
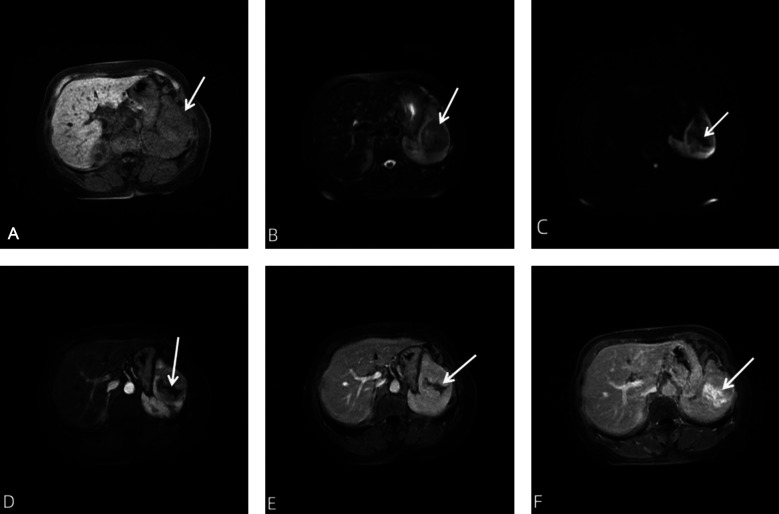
MRI of the patient's spleen. (**A**) Conventional spleen MRI scan showed a type of round mass in the spleen (white arrows). Most of the lesions on T1WI showed isointensity shadows, and localized slightly high signal shadows. (**B**) Most of the lesions on T2WI showed slightly low signal shadows, with strip-shaped high and low mixed signal shadows (white arrows). (**C**) In the DWI sequence, the overall signal was low, and lower signal shadow was seen inside (white arrow). (**D–F**) Contrast-enhanced MRI showed obvious enhancement in the arterial and venous phases of the lesion, with strip-like unenhanced and enhanced areas inside (white arrows) in delayed scan, and isointensity shadows in the remaining areas. DWI, diffusion-weighted imaging; MRI, magnetic resonance imaging; T1WI, T1-weighted imaging; T2WI, T2-weighted imaging.

Plain and contrast-enhanced CT scans of the spleen showed local enlargement of the spleen, with a type of circular isodensity and punctate calcifications (Figure 1). The lesions in the arterial and venous phases showed progressive enhancement, with a CT value of about 40 HU on the plain scan, and a CT value of about 84 HU in the enhanced part of the venous phase. Conventional and contrast-enhanced magnetic resonance imaging (MRI) scans of the spleen showed localized enlargement of the spleen with abnormal intracellular signal and well-defined lesions (Figure 2). T2-weighted imaging (T2WI) scans showed that most of the lesions were slightly low-intensity shadows, with strip-shaped high-low mixed signal shadows, while T1-weighted imaging (T1WI) showed that most of the lesions were iso-intensity shadows, with slightly high signal shadows in local areas. Diffusion-weighted imaging (DWI) showed hypointense shadows with stripes of lower signal shadows inside. The lesions showed obvious enhancement in arterial and venous phases, with strip-like unenhanced areas inside and internal strip-like enhancement in the delayed scan and the remaining areas showed isointensity shadows.

**Figure 3:**
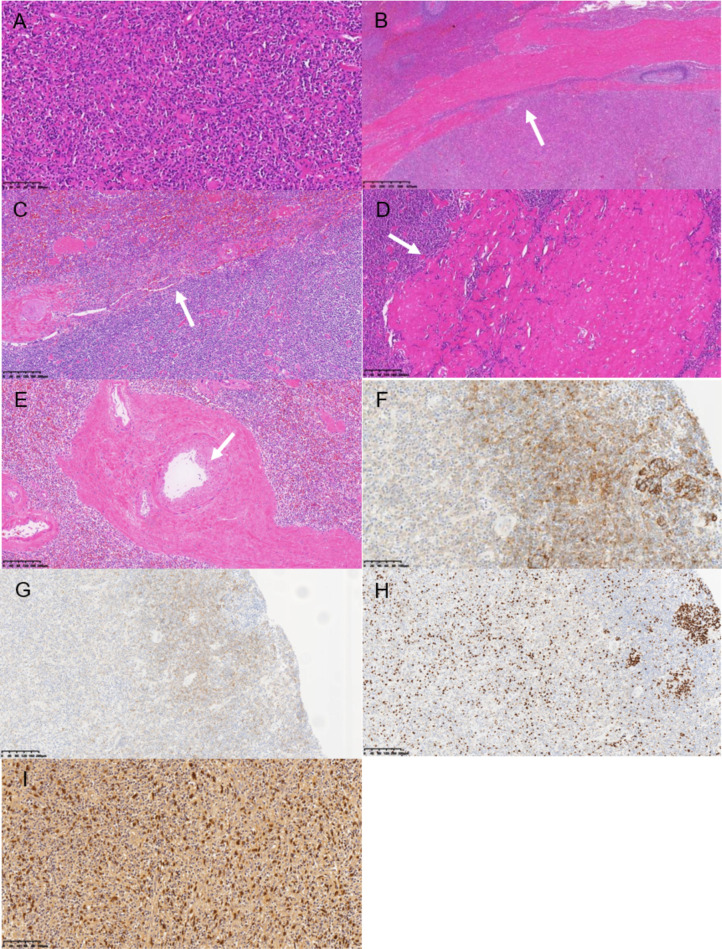
(**A**) The tumor cells were arranged in bundles and spirals, and the cells were oval. No mitoses were seen. There were scattered lymphocytes and plasma cells infiltrating HE-200. (**B**) Fibrous capsule (white arrow) HE-100 was visible at the tumor margin. (**C**) The tumor showed poorly defined borders and no capsule (white arrow) HE-100. (**D**) Intratumoral scar tissue (white arrow). (**E**) Thick blood vessels (white arrows) HE-100 were seen in the scar. (**F**) Tumor cells were positive for CD21 HE-21. (**G**) Tumor cells were positive for CD23 HE-100. (**H**) Tumor cells expressed 20%–30% Ki-67 HE-100. (**I**) Positive EBER expression was seen in tumor cells for HE-200.

Splenectomy was performed after the examination. The spleen was completely removed by surgery after the diagnosis of the splenic tumor by rapid pathology. A splenectomy specimen was obtained. A gray tumor with a diameter of about 5 cm was observed in the spleen section. HE-stained tumor cells were fascicular, swirled, oval cells, with no mitoses, with scattered lymphocyte infiltration. Immunohistochemistry showed CD21 (+), CD23 (+), CD35 (+), BCL-2 (−), Bcl-6 (−), CD10 (−), CD138 (−), CD15 (−), CD20 (−), CD3 (−), CD30 (−), CD43 (−), CD45RO (−), CD5 (−), CD79α (−), Cyclin D1 (−), Ki67 (∼20% +), Kappa (−), Lambda (−), MUM-1(−), PAX-5 (−), CKpan (−), CD31 (−), CD34 (−), β-Catenin (−), CD68 (partial +), Desmin (−), S-100 (−) and ALK(1A4) (−). In situ hybridization showed EBER positive (Figure 3). The final pathological diagnosis was inflammatory pseudotumoral splenic follicular dendritic cell sarcoma. The patient recovered well after surgery. No tumor recurrence was found during the 1-year follow-up.

## Discussion

Follicular dendritic cell sarcoma (FDCS) was first reported by Monda et al. in 1986 (7). FDCS is a rare low-grade malignant tumor that mainly occurs in the lymph nodes and occasionally in the liver and spleen. The morphology of FDCS is similar to that of inflammatory pseudotumor but different from classic FDCS. IPT-like FDCS was first reported in 2001 by CHEUK et al. (8). Its histopathology is different from classical FDCS and is closely related to EBV infection. Studies have shown that EBER-positive tumor cells were found by *in situ* hybridization in almost all cases (1). It is extremely rare, with a higher incidence in women than in men (9), and there are generally no specific clinical symptoms. The final diagnosis of this disease depends on pathological examination. The pathological results of the patient in this report showed that the tumor cells were fascicled and swirled, the cells were oval, and no mitoses were seen. There were scattered lymphocytes and plasma cells infiltrating the inflammatory background. These findings were consistent with a previous report (10) for IPT-like FDCS-specific behavior (11–14). Histologically, IPT-like FDCS is similar to inflammatory pseudotumor, which can easily lead to misdiagnosis. However, based on the immunohistochemistry of this case, the positive expression of follicular dendritic cell (FDC) markers CD21, CD23, and CD35 can be correlated with that of inflammatory pseudotumor. At the same time, *in situ* hybridization of EBER (+) and inflammatory cells are helpful to distinguish IPT-like FDCS from other FDCS subtypes (15–17).

Spleen IPT-like FDCS imaging findings are rarely reported (18). The majority of reported lesions are solitary, round, solid, or cystic-solid, with clear borders and occasional punctate calcifications. CT-enhanced lesions were mildly enhanced in the arterial phase, further enhanced in the venous phase, and showed continuous enhancement (19), which is consistent with the findings of the case presented herein. Bui reported the presence of a central stellate area in the lesion showing low signal intensity on T1 and T2-weighted MRI due to fibrosis and varying degrees of necrosis (18), unlike in our case, where intralesional fibrous scar tissue and thickened blood vessels were seen, which is the pathological basis for delayed enhancement on T2WI in enhanced MRI. This is a characteristic image that has not been previously reported. Our case showed different enhancements. The early enhancement was weaker than the spleen, a clear demarcation and the local capsule could be seen; the T2WI scan showed that most of the lesions were slightly low-signal shadows, with strip-shaped high-low mixed signal shadows inside. The high and low signals were caused by fibrous scars and vascular components, while DWI showed low signal shadows, with strip-shaped lower signal shadows inside, and fibrous scars explain the existence of low signal. Therefore, this case report described obvious characteristic manifestations. The lesions were round and solid, with delayed enhancement, and the enhancement degree was higher than that of the surrounding normal spleen parenchyma. The characteristic is called delayed enhancement of scars. The enhanced MRI scan should be delayed for at least three minutes to show the enhancement characteristics. The CT enhancement was not seen because the scanning time was not reached, and it was mistaken for cystic degeneration. The enhancement mode is the newly discovered important sign of this disease, which is helpful for future imaging diagnoses.

Based on the data published to date, splenectomy is the treatment of choice if the tumor is confined to the spleen. There is less recurrence or metastasis after surgery. Adjuvant therapy is not required (20). The patient in this report underwent complete tumor resection and was followed up for one year after surgery. The patient is currently healthy, with no tumor recurrence or metastasis.

Differentiation of spleen IPT-like FDCS from other splenic tumors is necessary (21). 1. Spleen inflammatory myofibroblastic tumor is also called inflammatory pseudotumor phase identification. Because inflammatory pseudotumor is composed of spindle cells and inflammatory cells, it lacks blood supply, with delayed enhancement performance, but it is always lower than that of the normal spleen. Parenchymal enhancement (22), imaging differences with splenic IPT-like FDCS. 2. Sclerosing hemangiomatous nodular transformation is a rare non-neoplastic vascular disease of the spleen. Concentric enhancement and spoke sign are considered to be characteristic features of SANT, while the central part of the lesion is delayed without enhancement (23), which can be differentiated from IPT-like FDCS. 3. Given that the splenic hamartoma is a solid mass with clear boundary, isointensity on T1WI and hyperintensity on T2WI, with heterogeneous enhancement in the early stage, and a similar signal compared to the surrounding spleen parenchyma in the later stage, it is not difficult to distinguish from IPT-like FDCS (21). 4. Spleen angiosarcoma must be considered because of the dismal prognosis of the disease. The CT manifestations of angiosarcoma are heterogeneous low-density shadows with unclear borders and round or oval shapes; most of them are solitary lesions, and a few can be multiple. Because the lesions are prone to hemorrhage, cystic areas are prone to form, and the enhanced scan looks like a hemangioma. It is enhanced from the edge of the lesion first, and then the contrast gradually fills to the center. MRI shows uneven low signal on T1WI, uneven high signal on T2WI, and high signal intensity on DWI. The enhancement is the same as CT appearance. Because it is a malignant tumor, there are metastases. Once intrahepatic metastases are found, the diagnosis of angiosarcoma is not difficult (24).

## Conclusion

IPT-like FDCS in the spleen is very rare, the clinical manifestations lack specificity, and no characteristic imaging signs were found in all previous reports. However, this study found that the tumor had delayed enhancement of scars, and the appearance of this characteristic manifestation was helpful for preoperative diagnosis. However, the final diagnosis should be made based on a pathological immunohistochemical examination.

## Contribution to the field statement

Follicular dendritic cell sarcoma (FDCS) is a rare lymphoid hematopoietic tumor of unknown etiology. It is composed of spindle-shaped and oval cells, mixed with a large number of inflammatory cells, and the typical fascicular and spiral arrangement is rare, similar to inflammatory pseudotumor, so it is described as inflammatory pseudotumor-like follicular dendritic sarcoma(IPT-like FDCS), a low-grade malignancy, which has been shown to be associated with EBV, pathologically characterized by positive expression of immune markers including CD21, CD35, and CD23, which is rare and involves the spleen The reports on the imaging manifestations of IPT-like FDCS are all case reports. Due to the lack of specific clinical manifestations, preoperative diagnosis often leads to misdiagnosis. Reviewing the literature and imaging reports that the disease lacks imaging characteristics, we provide this case, which is confirmed by pathology as spleen IPT-like. FDCS, through the patient's clinical, imaging and pathological data, summarizes the imaging features and compares them with the pathology, and finds a new characteristic imaging sign, that is, the characteristic of scar enhancement in the lesions in the delayed MRI enhanced scan. At the same time, we found that the pathology There are fibrous and vascular structures inside the lesion. Through our case report, the accuracy of preoperative diagnosis of this disease can be improved for future clinical work.

## Data Availability

The original contributions presented in the study are included in the article/Supplementary Material, further inquiries can be directed to the corresponding author/s.
